# Hyperparameter Optimization Method Based on Harmony Search Algorithm to Improve Performance of 1D CNN Human Respiration Pattern Recognition System

**DOI:** 10.3390/s20133697

**Published:** 2020-07-01

**Authors:** Seong-Hoon Kim, Zong Woo Geem, Gi-Tae Han

**Affiliations:** 1 Department of Computer Engineering, Gachon University, Seongnam 13120, Korea; limiteddaily@gmail.com; 2 Department of Energy IT, Gachon University, Seoongnam 13120, Korea; geem@gachon.ac.kr

**Keywords:** 1D convolutional neural network, ultra-wideband radar, respiration patterns, harmony search algorithm, hyperparameter optimization

## Abstract

In this study, we propose a method to find an optimal combination of hyperparameters to improve the accuracy of respiration pattern recognition in a 1D (Dimensional) convolutional neural network (CNN). The proposed method is designed to integrate with a 1D CNN using the harmony search algorithm. In an experiment, we used the depth of the convolutional layer of the 1D CNN, the number and size of kernels in each layer, and the number of neurons in the dense layer as hyperparameters for optimization. The experimental results demonstrate that the proposed method provided a recognition rate for five respiration patterns of approximately 96.7% on average, which is an approximately 2.8% improvement over an existing method. In addition, the number of iterations required to derive the optimal combination of hyperparameters was 2,000,000 in the previous study. In contrast, the proposed method required only 3652 iterations.

## 1. Introduction

Existing ultra-wideband (UWB) radar sensors have been used for shortrange communication. However, recently, many studies using this sensor have been conducted in various fields that require noncontact signal pattern analysis technology. Among them, in the healthcare field, a technique to analyze the bio-signal data of a person obtained using a UWB sensor is being actively researched. This technology is intended to analyze signal patterns, e.g., the respiration, pulse, and movement of a person, obtained from a UWB sensor and use these signal patterns to diagnose diseases or improve quality of life based on the analyzed data.

Various machine learning techniques have been studied as a method to recognize and analyze human bio-signal patterns. Recently, as deep learning technologies have been researched, attention has been placed on improving performance, e.g., by improving the recognition rate [1,2,3,4,5].

Previously, the pattern recognition method for UWB sensor signals was researched primarily on pulse or apnea pattern detection based on machine learning [6,7,8,9,10]. Since then, as ANN(Artificial Neural Network)-based AI(Artificial Intelligence) technology has emerged, methods using this technique have been investigated; however, in such methods, preprocessing steps are required to construct learning data of a specific pattern. Recently, deep learning models based on CNN have emerged to solve these problems.

In such methods, features can be extracted and learned in the same process without preprocessing the data. Thus, various pattern recognition studies using CNN models have been conducted recently [11,12,13]. 

Generally, a CNN is used for object detection and recognition from an image; thus, it primarily comprises input data structures in two dimensions; however, one-dimensional signal data can also be used as input. Therefore, when one-dimensional signal data are input, convolution is performed using a one-dimensional vector kernel. The respiratory signal of a human obtained from a UWB sensor is also one-dimensional data; therefore, it is possible to learn and recognize the patterns of respiratory signal by implementing a 1D CNN model. However, generally, CNN performance is greatly affected by the structural design of each layer and hyperparameter values.

Various image processing studies have demonstrated that it is possible to improve performance with little adjustment of the depth and structure of the layer and the number and size of kernels in each layer based on previously studied neural networks and hyperparameters. However, in fields with few related studies, it is necessary to design an optimal neural network via repeated experiments with various hyperparameter combinations using a simple neural network [14,15,16,17]. 

In previous 1D CNN-based respiration pattern recognition studies, a specific range for hyperparameters is determined in advance for each layer, and experiments are performed repeatedly using a combination of all hyperparameters in this range, and the best combination is selected. In this case, the total number of hyperparameter combinations is 2,000,000; however, this method is limited relative to finding a hyperparameter with better performance because the combination is generated using specific values in the specified range.

To find a combination of hyperparameters that yields improved performance, the search must be performed in a wider range. In this case, experimenting with all combinations is very inefficient because it takes a lot of time. Therefore, it is necessary to study how to find an optimized combination of hyperparameters that provides good performance in less time. There are various algorithms for the optimization method, but among them, the harmony search (HS) algorithm is designed with a structure that is easy to hybridize with other technologies than the traditional optimization methods, namely the genetic algorithm (GA) or particle swarm optimization (PSO), and is an algorithm that efficiently searches for solutions [18,19,20,21,22,23,24].

Therefore, in this paper, we propose a hyperparameter optimization method that yields better performance in less time than the existing method [25] by applying the HS algorithm to the existing 1D CNN model with UWB radar sensor for respiration pattern recognition. A comparative analysis demonstrates that the proposed method outperforms existing methods.

## 2. Related Research

### 2.1. UWB Sensor and Repiratory Signal Patterns

UWB sensors have primarily been used for shortrange wireless communication; however, recently, they have been widely used for object motion detection and bio-signal detection.

The UWB sensor uses a wide range of frequency bands; thus, it has the advantage of less interference with other narrowband or wideband signals.

In particular, UWB signals use very narrow pulses, i.e., a few nanoseconds or picoseconds. Therefore, it is possible to precisely measure the distance and position of an object with a low-spectrum power density characteristic in a wide frequency band.

The specifications of the UWB sensor used in our experiment are given in Table 1, and the data were measured via UART communication with a PC.

The respiratory signals of the UWB sensor rise in inspiration and descend in expiration. The amplitude of the respiratory signal is determined by the degree of deep respiration, and, as the length of one respiration lengthens, the period of the signal increases [25,26,27,28].

These respiratory signals appear in various forms depending on the type of respiration, and the medical field defines the respiration type as given in Table 2 based on the number of respirations per minute [29,30,31].

**Figure 1:**
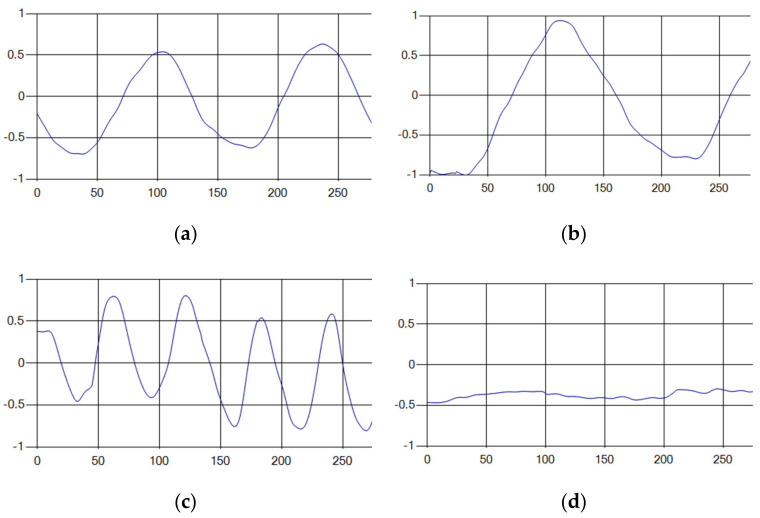
Four respiratory signal patterns measured by UWB sensor. (**a**) Eupnea. (**b**) Bradypnea. (**c**) Tachypnea. (**d**) Apnea.

Each respiration type is measured using a UWB sensor (Figure 1), and each signal demonstrates a distinct difference.

### 2.2. 1D CNN for Signal Pattern Recognition

**Figure 2:**
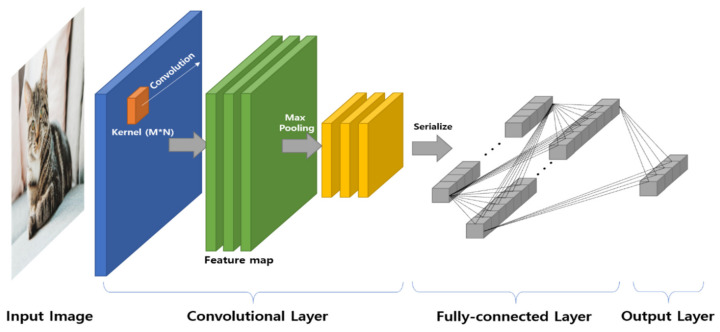
General structure of CNN for image classification.

Generally, CNNs are used for object detection or recognition based on image data. Thus, as shown in Figure 2, the convolutional layer uses an M × N type kernel for feature extraction of the image. In addition, features extracted by performing convolution with multiple kernels are processed into vector form and connected to the fully-connected layer.

In addition, this layer is connected to the output layer, and through an iterative learning process, a model that can derive the classification result is constructed.

The respiratory signal comprises a one-dimensional signal (rather than an image) to learn respiratory patterns using an existing CNN. Therefore, the respiratory signal must be transformed into two-dimensional image data. However, since one-dimensional data have time continuity, if the data are transformed into two-dimensional data, the time continuity characteristic for a respiratory signal may be impaired.

In addition, the structure of one-dimensional data changes to a matrix. As a result, unspecific features may be added between each row in the convolution result. Due to these problems, the performance of the neural network can be degraded. Thus, it is necessary to construct a CNN that is suitable for one-dimensional data characteristics.

**Figure 3:**
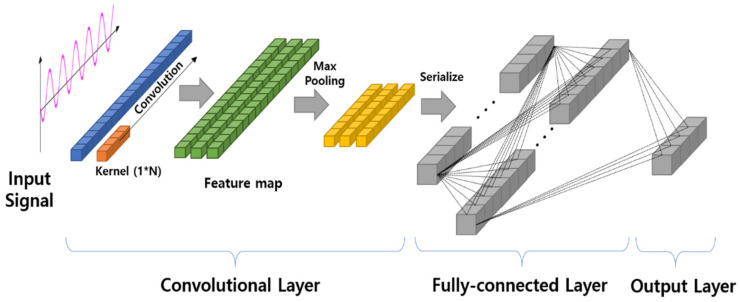
General structure of 1D CNN for signal pattern recognition.

A CNN for learning the one-dimensional data is shown in Figure 3. Here, the convolutional layer receives input data in the form of a vector and performs convolution using a 1 × N type kernel. As a result, the feature map is also extracted in the form of one-dimensional data. In the subsequent learning process, the fully-connected layer is structured in the same manner as a general CNN, and learning is performed via backpropagation.

As described above, a CNN can be designed with a structure to learn 2D and 1D data, and the optimization model is completed through feedforward and backpropagation processes. For learning via backpropagation, learning proceeds with various optimization techniques. Generally, stochastic gradient descent (SGD) based on the gradient descent method is employed.

Recent deep neural networks use several optimization methods to improve SGD. Such methods attempt to minimize errors for various hyperparameters and learn the data of a given neural network. Thus, the hyperparameter settings are largely determined by existing studies or experiments.

### 2.3. Hyperparameters in 1D CNN

The hyperparameters in a CNN can be composed of various components depending on the structure of the neural network. The basic hyperparameters are shown in Table 3. Here, kernel size and kernel count are the most influential factors relative to recognition or detection performance. The hyperparameters other than the kernel size and kernel count in Table 3 are already reported the ranges of values close to optimization through various studies [32,33,34].

However, the kernel size and count should be applied differently depending on the characteristics of the input data. Thus, the kernel size and count must be found through repeated experiments or experience.

Methods to optimize these hyperparameters have been studied in various ways. The basic optimization methods are summarized as follows.

- Manual search: This method sets the hyperparameter value depending on the researcher’s intuition or experience and can be used when the understanding of neural network structure and learning data is high. However, this method is inefficient because hyperparameter setting criteria are very ambiguous and many experiments are required.
- Grid search: This method finds the hyperparameter that yields the best performance by predetermining several values for each hyperparameter and combining the values.
- Random search: This method finds the best combination by setting the minimum and maximum range of values that each hyperparameter can have and selecting values randomly within the specified range. This method can be better than the manual search and grid search methods in terms of performance over time.
- Bayesian optimization: Bayesian optimization constructs a specific range of value for a hyperparameter based on a good case studied in the past and optimizing within the determined range. This method has good performance over time but must be studied previously.

In the existing study of respiration pattern recognition using the UWB sensor signal, the 1D CNN hyperparameters were optimized using the grid search method. However, to improve the neural network’s performance, a better method to optimize hyperparameters is required. The Bayesian optimization method, which demonstrates good performance, can be used when existing studies are abundant; however, for 1D CNN-based respiration pattern recognition, it is difficult to use this method due to a lack of related studies. The random search method can also take a very long time because it optimizes the values randomly selected for given ranges without prescribed rules.

### 2.4. Harmony Search Algorithm

The harmony search (HS) algorithm solves optimization problems based on stochastic theory. This algorithm solves such problems by modeling the process for the music-inspired player to produce better chords.

The HS and genetic algorithms are similar relative to the method by which they probabilistically approach the solution through a random process; however, they differ in the process of constructing the initial value group. The HS algorithm comprises five processes, i.e., the initial value setting, harmony memory (HM) initialization, new harmony creation, HM update, and repetition processes [35,36].

Step 1: Initial value setting

To optimize the hyperparameters used in the HS algorithm, initial values must be set for the hyperparameters. Here, the HS algorithm has three parameters, i.e., harmony memory size (HMS), harmony memory considering ratio (HMCR), and pitch adjusting ratio (PAR). In addition, an objective function is required. The objective function is used to evaluate the quality of various chords, and the optimization problem is solved by maximizing or minimizing the value of the objective function.

Step 2: Initialize harmony memory

Harmony memory is used by the player to find the best chord among multiple chords, and one chord comprises several variables. The number of initialization chords is configured to have the HMS, and the objective function is set to evaluate the chord. During initialization of HM, the values of each variable are determined by random selection. Here, the value is selected in the range of the random selection for each variable. The objective function is calculated by the values of the variables comprising harmony, and the HM is updated with the values of the variables and the objective function.

Step 3: Create new harmony

This process removes bad chords from HM and finds better chords. Here, the HS optimization hyperparameter HMCR is used to determine whether to create a completely new chord or make a chord by slightly adjusting the existing chords. The HMCR is set to a real value between 0 and 1, and, if this value is too low, new chords are created frequently, which can be result in an inefficient optimization process. Conversely, if the HMCR value is too high, the existing chord is selected frequently and searched around it; thus, there is a risk of falling into the local minimum or maximum. Therefore, it is necessary to find a suitable HMCR to ensure sufficient performance of the HS algorithm. When using an existing chord in the HMCR, it is necessary to adjust the pitch, which is determined by hyperparameter PAR, which is set to a real value between 0 and 1. Here, if the value is large to be close to 1, it is used by changing the existing chord. In addition, when the pitch is corrected by PAR, the amount of change is adjusted by the maximum pitch adjustment proportion (MPAP) and maximum pitch adjustment index (MPAI) values. MPAP is a pitch adjustment value used for discrete variables, and MPAI is a pitch adjustment value used for continuous variables. Pitch adjustment values serve to search around existing chords. If the value is small, a local search is performed, and if it is large, a wide range is searched.

Step 4: HM update

For chords newly created by HMCR and PAR, the objective function value is calculated for comparison with other chords. Then, the objective function value of the new chord is compared to the worst chord of the HM composed so far. Here, if the new chord is better than HM’s worst chord, the bad chord is replaced by the new chord.

Step 5: Iteration and results

Steps 3 and 4 are performed as many times as the set number of iterations, and optimization is finished by selecting the chord that demonstrates the best performance in the final HM. The performance of the HS algorithm is ultimately determined by the values of HMS, HMCR, PAR, MPAP, and MPAI. These five variables should also be set to appropriate values in reference to existing studies or experiments.

The HS algorithm has five hyperparameters to be set. Thus, it is possible to reduce the number of variables to be tested and effectively find the solution.

Therefore, this paper proposes an optimization method for 1D CNN hyperparameters based on the HS algorithm and demonstrates the superiority of the proposed method by applying it to existing 1D CNN respiration pattern recognition system.

## 3. Proposed Method

This paper proposes a method to optimize hyperparameters to improve the recognition rate in a 1D CNN neural network configured to recognize respiratory signal patterns from UWB sensor data. In the proposed method, first, the required hyperparameters are set as target variables to be optimized, and HM comprises hyperparameters and objective functions.

Next, the parameters to be used by the HS algorithm are derived, and in the final step, the optimized hyperparameter combination is found by applying the HS algorithm to the 1D CNN respiration pattern recognition system.

The goal of the proposed method is to design an optimal system that integrates the 1D CNN and HS algorithms in order to find an optimal combination of hyperparameters that yields an excellent respiratory pattern recognition rate in the 1D CNN system.

To demonstrate that the proposed method is superior to the existing method, an experiment was conducted under the same conditions as the existing method, and the respiratory signal pattern recognition rate and number of iterations required to find the optimal combination of hyperparameters were compared.

### 3.1. Design of Harmony Memory and Object Function

To optimize the 1D CNN’s hyperparameters using the HS algorithm, first, the HM must be configured and the objective function must be designed. Here, hyperparameters that significantly influence optimization of the 1D CNN are selected. The hyperparameters used in the proposed method are presented in Table 3, and these hyperparameters affect the recognition rate according to the characteristics of the input data. The hyperparameters selected for the proposed method are KS, KC, and DLNC (Table 4), where KS is the kernel size in the convolutional layer, KC is the number of kernels used in the convolutional layer, and DLNC is the number of neurons in the dense layer.

KS and KC exist for each convolutional layer. Thus, if the convolutional layer depth (CLD) increases, KS and KC will also increase.

In the 1D CNN, the dense layer always comprises two layers regardless of the CLD.

**Figure 4:**
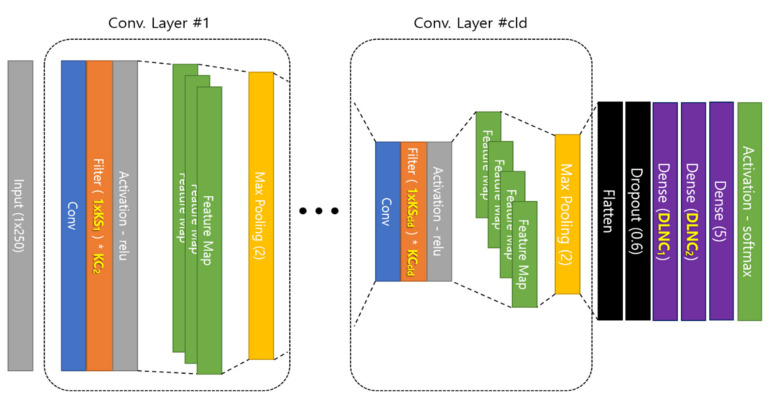
Layout of 1D CNN model used in proposed method.

The HM is designed to have the convolutional layer as much as CLD (Figure 4) for the existing 1D CNN respiration pattern recognition system. As the value of CLD increases, the number of hyperparameters for KS and KC also increases, and the number of DLNC is fixed to two.

The structure of the hyperparameters of HM, i.e., the optimization target, is expressed in matrix form as follows.

$$HM=\left[\begin{array}{cccccccc} ks_{1}^{1}\; & kc_{1}^{1}\; & \ldots \; & ks_{1}^{cld}\; & kc_{1}^{cld}\; & dlnc_{1}^{1}\; & dlnc_{1}^{2}\; & R_{1}\\ ks_{2}^{1}\; & kc_{2}^{1}\; & \ldots \; & ks_{2}^{cld}\; & kc_{2}^{cld}\; & dlnc_{2}^{1}\; & dlnc_{2}^{2}\; & R_{2}\\ ks_{3}^{1}\; & kc_{3}^{1}\; & \ldots \; & ks_{3}^{cld}\; & kc_{3}^{cld}\; & dlnc_{3}^{1}\; & dlnc_{3}^{2}\; & R_{3}\\ & & & & \ldots & & & \\ ks_{hms}^{1}\; & kc_{hms}^{1}\; & \ldots \; & ks_{hms}^{cld}\; & kc_{hms}^{cld}\; & dlnc_{hms}^{1}\; & dlnc_{hms}^{2}\; & R_{hms} \end{array}\right]$$

$$HP_{i}=\left\{ks_{i}^{1},\;kc_{i}^{1},\;\ldots ,\;ks_{i}^{cld},\;kc_{i}^{cld},\;dlnc_{i}^{1},\;dlnc_{i}^{2}\right\},\;i=1,\;\ldots ,\;hms$$

The objective function used to evaluate the performance of each hyperparameter combination in HM is set to recognition rate $R_{i}$.

**Figure 5:**
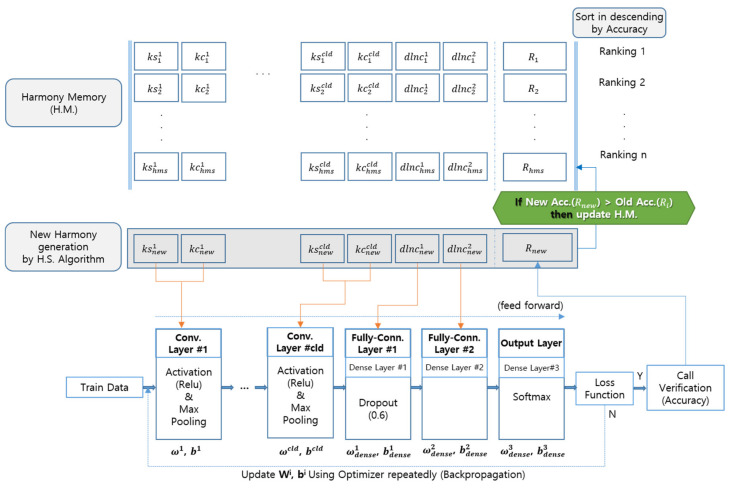
Process for hyperparameter optimization between HM and 1D CNN.

In other words, in each execution step of the 1D CNN (Figure 5), hyperparameters are substituted, and weights ω and bias b are updated while the feedforward and backpropagation processes are performed with the learning data. Learning for a single selected parameter combination is completed when the loss function’s conditions are satisfied.

Next, the recognition rate using the verification data is calculated and stored at the location of the objective function in the new HM area ($HM_{new}$), and the HM is updated by comparing the $R_{new}$ of $HM_{new}$ to $R_{hms}$ of HM, which is the lowest priority of HM. When HM is updated, alignment is performed for recognition rate $R_{i,i=1,\ldots ,hms}$ in HM.

When initializing HM, it is recommended to set HMS to a sufficiently large value (in this study, it is set to 1000). The reason may be described as an example of searching global maxima, which has the highest recognition rate for several CNN hyperparameters.

If a global search is performed through multiple random selections during the HM initialization process, and iteration is performed based on this HM, the HM creation with sufficiently large HMS can be optimized with much fewer Iterations than when HMS is small. Thus, the initialization process requires more time than when HMS is small; however, it is possible to significantly reduce the time required to reach global maxima by iteration. In contrast, if a small HMS value is used, the memory pool will also be small; thus, there is a high probability of falling into local maxima or performing a very high number of iterations.

### 3.2. Selecting Optimal Hyperparameters for HS Algorithm

The HS algorithm requires hyperparameters to operate HS itself in addition to optimization for a 1D CNN, and, to obtain good results, the hyperparameters should be selected with appropriate values determined through experimentation. Therefore, experiments were performed by constructing various hyperparameter groups, and appropriate hyperparameters were set based on the results. The hyperparameters that have significant influence on the performance of the HS algorithm in the proposed method are HMCR, PAR, and MPAP. Here, as the HMCR value increases, the probability of using a combination of existing hyperparameters stored in HM increases. Pitch is adjusted by PAR, and a higher PAR value results in a higher probability of pitch adjustment. Here, pitch is adjusted by selecting a random value within the MPAP range. In other words, a higher HMCR value results in a higher probability that the PAR and MPAP values will be used frequently. Therefore, HMCR should be set to an appropriate value to achieve efficient optimization without reaching local maxima. Conversely, if the value of the HMCR is low, optimization efficiency is reduced because this is similar to global search using normal random selection [37].

Considering the characteristics of each hyperparameter, we present two cases where optimization becomes inefficient with the local maxima or frequent random selection in the following.

1. Local maxima problem: This problem occurs when using a high HMCR value and low MPAP value regardless of the PAR value. This problem frequently recalls existing memory due to the high HMCR value, and the pitch is adjusted by PAR when creating a new harmony. Here, if the MPAP value is too low, falling into local maxima may occur.
2. Random selection problem: This problem occurs when the HMCR value is low. As a result, the probability of random selection occurring increases, which can yield efficiency that is similar to a typical random search method. This problem may also occur when the HMCR, PAR, and MPAP values are all high. In particular, if MPAP is too large, even if the existing HM is used, the pitch adjustment value becomes too large, which has the same effect as random selection.

Due to these problems, it is inefficient to use HS algorithm variables that are close to 0 or 1. To minimize this problem, HMCR is set to a random value of 0.05 units between 0.5 and 0.7. Thus, when creating a new harmony, it is recommended to recall the existing HM with a probability of 50% or greater.

In addition, PAR was selected with a random value of 0.05 units in the range of 0.6 to 0.8 such that pitch adjustment occurred frequently. Here, the PAR value is set to a slightly higher value than existing method 2 in Table 5. Thus, MPAP must be set to an appropriate value to minimize the occurrence of local maxima and random selection problems.

The MPAP value was set in the range 10–18 through experiments. The hyperparameter combination and performance of the HS algorithms among studies are compared in Table 5. Note that the performance comparison is expressed as the number of iterations required to reach a 95% recognition rate for each combination of hyperparameters.

As can be seen, the hyperparameter combination of the proposed method reached a recognition rate of 95% with fewer iterations than the hyperparameter combination used in other studies. The hyperparameter values for the existing method 1 and 2 shown in Table 5 are used in different optimization problems [38,39].

As shown in Table 5, when applying the combination of hyperparameters derived from existing methods 1 and 2 to the hyperparameter optimization problem of the proposed ID CNN, approximately 4,357 to 5,912 iterations were required reach the target recognition rate of 95%. When the proposed method was applied to the same environment, it was possible to derive a combination of hyperparameters that reached the target recognition rate by performing approximately 2,011 iterations. Since MPAP is applied to all hyperparameters used in the proposed 1D CNN (i.e., KS, KC, and DLNC), a range of hyperparameters was derived experimentally to have a larger value than the hyperparameters suggested by the existing method. This means that each hyperparameter must be set appropriately for the target optimization problem. The experimentally derived HMCR, PAR, and MPAP values were used in the HS algorithm to optimize the KS, KC, and DLNC hyperparameters of the proposed 1D CNN.

### 3.3. Optimization of 1D CNN Hyperparameters Using HS Algorithm

**Figure 6:**
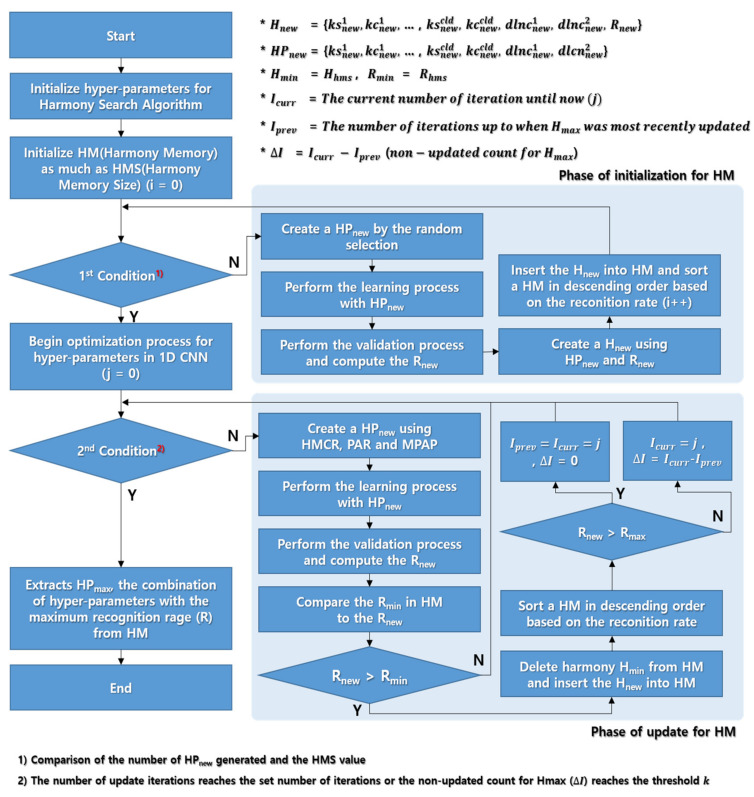
1D CNN hyperparameter optimization process using HS algorithm.

Figure 6 shows the hyperparameter optimization process to improve the recognition rate of respiration patterns in the 1D CNN using the HS algorithm with the HM configuration suggested in Section 3.1 and the HS hyperparameter range suggested in Section 3.2.

The new harmony generation and harmony memory update processes shown in Figure 6 are detailed in Algorithm 1. Here, a combination of hyperparameters as much as HMS is generated and filled in HM. Then, the HM was updated only when the given condition was satisfied. $H_{max}$ is a combination of hyperparameters that gives the best performance among the harmonies in HM. If the HM is updated but $H_{max}$ has not been updated, variable ΔI (count of non-update for $H_{max}$) is increased by one. If the ΔI value reaches a given specific value (threshold k), optimization is no longer occurring in HM, and the HM update operation is completed even if the set number of iterations has not been reached.

Finally, the hyperparameter combination $HP_{max}$, which has the highest recognition rate in HM, is derived as the optimal combination of hyperparameters.
**Algorithm 1:** Algorithm for Optimizing Hyperparameters of 1D CNN using HS(1)Set the parameters for Harmony Search (HS) algorithm$HMS=1000,HMCR=rand\left(0.5,\;0.7\right),\;\;PAR=rand\left(0.6,\;0.8\right),\;MPAP=rand\left(10,\;18\right),$k=threshold for optimization completion condition*KS-MIN*=minvalueof kernel size,  *KS-MAX*=max value of kernel size*KC-MIN*=minvalueof kernel count,  *KS-MAX*=max value of kernel count*DLNC-MIN*=minvalueof dense layer neuron count*DLNC-MAX*=maxvalueof dense layer neuron count (2)Initialization of Harmony Memory (HM).    Begin Loop (i):          for(i = 0; i < HMS; i++)①Set the value of each hyperparameter $HP_{i}\;$ through random selection     Begin Loop (d):        for (d = 1; d <= CLD; d++)              $ks_{i}^{d}=rand$(KS-MIN, KS-MAX)            $kc_{i}^{d}=rand$(KC-MIN, KC-MAX)      End Loop (d)      $dlnc_{i}^{1}=rand$(DLNC-MIN, DLNC-MAX)      $dlnc_{i}^{2}=rand$(DLNC-MIN, DLNC-MAX) ②Learn with the hyperparameter $HP_{i}.$* Use training data set X* Update w, b by backpropagation        ω, b ← $L\left(\;HP_{i},\;X\right)$ ③If the Loss function is not satisfied, go to ②. ④Calculate the recognition rate $R_{i}\;$ by applying $HP_{i}\;$ to the verification data Y.      $R_{i}$ ← $V\left(\omega ,\;HP_{i},\;Y\right)$ ⑤Store the values of hyperparameters $HP_{i}$ and recognition rate $R_{i}$ in the i index of HM End Loop (i) (3)Optimization of 1D CNN Hyperparameter (HP).Initialization of variable $I_{prev}\;,I_{curr}\;(I_{prev}=0,I_{curr}=0$)Begin Loop (j):   for(j = 0; j<Iteration; j++)     ①Each algorithm is executed according to the following conditions  if rand(0, 1) < HMCR then     * Create new harmony $HP_{new}$ using the hyperparameter $HP_{rand}\;$ randomly       selected from the existing HM.            $HP_{rand}=HP_{i},\;i=\mathrm{rand}\left(0,\;HMS\right)\;and\;HP_{i}\in HM$            $HP_{rand}=\left\{ks_{rand}^{1},\;\;kc_{rand}^{1},\;\;\ldots \;,\;\;ks_{rand}^{cld},\;\;kc_{rand}^{cld},\;\;dlnc_{i}^{1},\;\;dlnc_{i}^{2}\right\}$         if rand(0, 1) < PAR then          * Adjust the pitch for the new harmony $HP_{new}$ as follows.           Begin Loop (d):               for (d = 1; d <= CLD; j++)                 $ks_{new}^{d}=ks_{rand}^{d}+MPAP$                 $kc_{new}^{d}=kc_{rand}^{d}+MPAP$           End Loop (d)           $dlnc_{new}^{1}=dlnc_{rand}^{1}+MPAP$           $dlnc_{new}^{2}=dlnc_{rand}^{2}+MPAP$        else if          * Create a new harmony without adjusting the pitch of the existing harmony.                                             $HP_{new}=HP_{rand}$  else if    * Set values for each hyperparameter $HP_{new}\;$ through Random Selection.        Begin Loop (d):           for (d = 1; d <= CLD; j++)              $ks_{new}^{d}=rand$(KS-MIN, KS-MAX)              $kc_{new}^{d}=rand$(KC-MIN, KC-MAX)      End Loop (d)      $dlnc_{new}^{1}=rand$(DLNC-MIN, DLNC-MAX)      $dlnc_{new}^{2}=rand$(DLNC-MIN, DLNC-MAX)  ②  Learn with the new harmony $HP_{new}.$    * Use training data set X.    * Update w, b by backpropagation                    ω, b ← $L\left(\;HP_{new},\;X\right)$  ③If the Loss function is not satisfied, go to ②.  ④Calculate the recognition rate $R_{new}\;$ by applying $HP_{new}$ to the verification data Y.                    $R_{new}$ ← $V\left(\omega ,\;HP_{i},\;Y\right)$  ⑤Compare the recognition rate $R_{new}$ with $R_{min}$, the minimum recognition rate in harmony memory. And if $R_{new}$ is greater than $R_{min}$, remove $H_{min}$ from harmony and update to $H_{new}$.  ⑥After sorting all harmony $H_{i}$ in descending order based on the recognition rate R of HM, perform the following procedure to find the optimal combination of hyperparameters early.   **if** $R_{new}>R_{max}$ then       $I_{prev}=I_{curr}=j$, ΔI=0   **else**         $I_{curr}=j$, $\Delta I=I_{curr}-I_{prev}\;$         **if** ΔI≥k then              break Loop (j)         **else**            continue Loop (j)End Loop (j) (4)Obtain the hyperparameter combination $HP_{max}$ with the highest recognition rate (R) from the harmony memory HM.

## 4. Experiments

### 4.1. Learning and Test Dataset

The UWB respiration signal dataset used for learning in this study is the same dataset used in a previous study to compare the performance to the exiting method [35].

The dataset includes five signal patterns, i.e., general respiration, slow respiration, tachypnea, apnea, and movement. Here, 10 people participated in the experiment. Table 6 shows samples of the respiratory signal data extracted for each pattern type.

The experimental data comprised a total of 2500 pieces (i.e., 10 peoples × 5 types × 50 pieces) by extracting 50 pieces for each type from each participant’s respiratory signal and 1 piece is a respiratory signal for 10 seconds. Here, 1500 pieces were used for learning, and the remaining 1000 were used for testing. In addition, 1500 pieces were divided into training and verification datasets at a ratio of 8:2. The two datasets comprised randomly selected data from 1500 data for each learning process.

### 4.2. Comparison of Recognition Rate between Proposed and Existing Methods

The range of hyperparameters used in the HS algorithm and 1D CNN for the proposed method were set as shown in Table 7. Here, KS, KC, and DLNC were approximately two times wider than in the previous studies.

For the experiment with the proposed method, the hyperparameters for the HS algorithm and 1D CNN were set as shown in Table 7, and number of iterations was set to 10,000 to perform repeated experiments. Here, if we calculate the number of all cases of the combination in the range of KS^1^, KS^2^, KS^3^, KC^1^, KC^2^, KC^3^, DLNC^1^, and DLNC^2^ used as hyperparameters of 1D CNN, the number of KS^i^ cases is 40 (odd numbers from 3 to 81), KC^i^ is 1009 (16 to 1024), and DLNC^i^ is 3841 (256 to 4096), so the total number of cases is 9.69934 × 10^20^ (= 40^3^ × 1009^3^ × 3841^2^).

**Figure 7:**
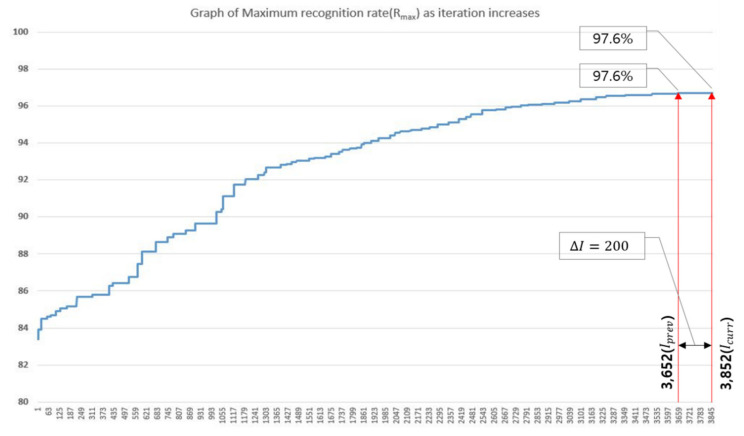
Trend of recognition rate in 1D CNN hyperparameter optimization process using proposed method.

Figure 7 shows the process of optimizing the hyperparameter combination of the 1D CNN. As can be seen, the recognition rate gradually is increased as iteration progressed. For $HP_{max}$, the recognition rate reached 96.7% after 3652 iterations, and optimization was terminated after 3852 iterations because it had not been updated continuously for over ΔI (200 iterations) repetitions.

The combination of hyperparameters and recognition rate $R_{i}$ for each iteration step is shown in Table 8, and it is possible to derive the optimal hyperparameter combination $HP_{max}$ at the location (i.e., recognition rate of $H_{max}$ has maximum values) where optimization was completed.

Comparing the above result with the existing method (Table 9), the final recognition rate and total number of iterations performed until searching for the optimal hyperparameter can be represented. In the existing method, a set of hyperparameters was constructed using a grid search method, and the recognition rate was tested for 2,000,000 combinations to optimize hyperparameters.

However, with the proposed method, it was possible to obtain a better recognition rate result in only 3652 iterations. In addition, with the existing method, the values of each optimized hyperparameter used in the 1D CNN were derived in a specific range. However, with the proposed method, each optimized hyperparameter was derived as a single value.

**Figure 8:**
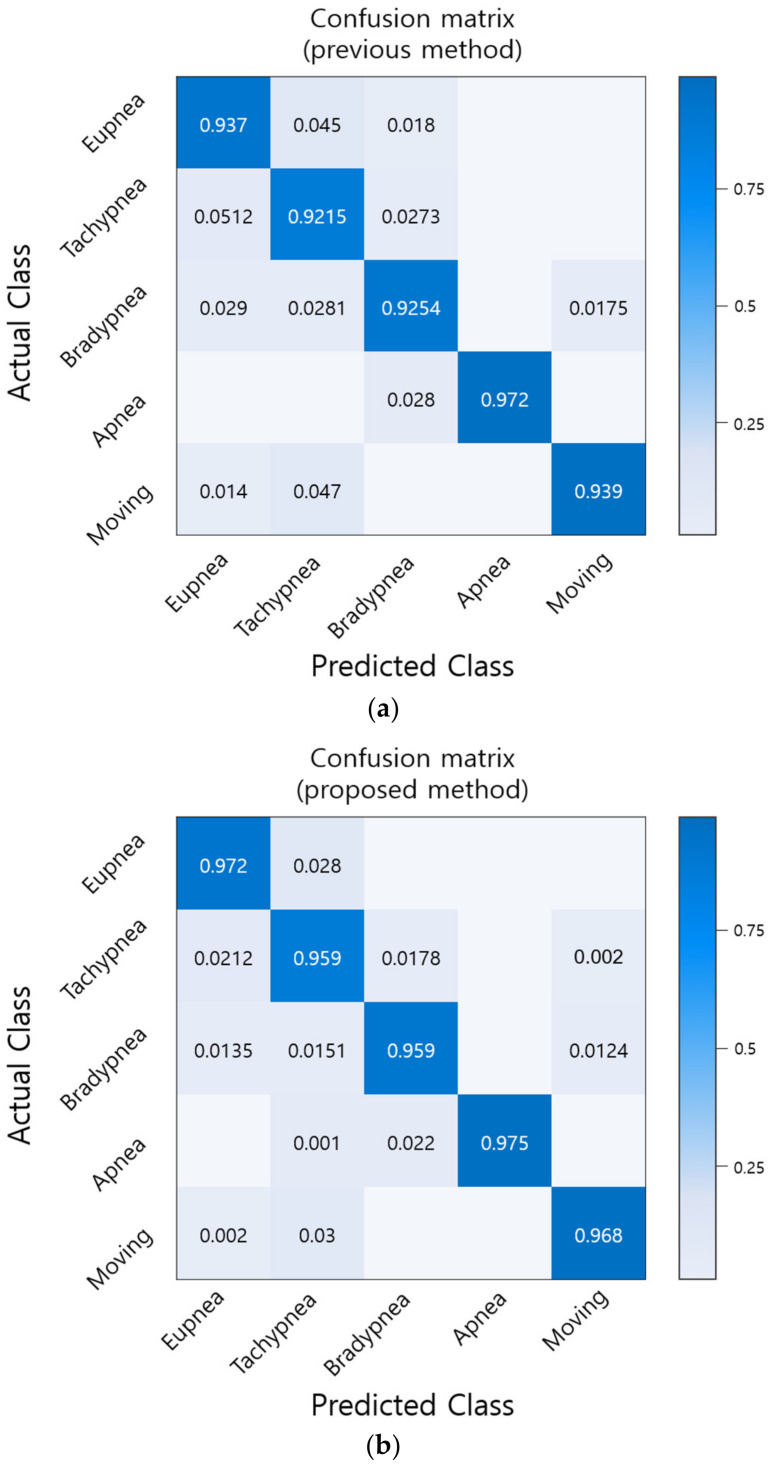
Comparison of recognition rate by respiration pattern for existing and proposed methods. (**a**) Recognition rate of existing method [28]; (**b**) Recognition rate of proposed method.

The recognition rate of 1000 test datasets was tested with optimized hyperparameters and compared to the existing method. The results are shown in Figure 8. By comparing all patterns, we found that the proposed method (Figure 8b) increased the recognition rate by approximately 2.8% compared to the existing method (Figure 8a).

## 5. Conclusions

In this paper, have proposed a method to optimize the hyperparameters required to improve the recognition rate in a 1D CNN-based UWB respiration pattern recognition study, and we have proven the validity of the proposed method by comparing it with an existing method. Conventionally, to optimize the hyperparameters of a 1D CNN, a specific range is determined for each hyperparameter, and a combination of the range values is constructed. Then, repeated experiments are performed using the combination in all cases. However, this method requires many experiments without special rules to find a better hyperparameter. Thus, it takes a long time to find the optimal hyperparameter combination, which is tedious and inefficient.

In contrast, the proposed hyperparameter optimization method is implemented to have scalability according to the CLD by integrating the HS algorithm into the 1D CNN and focusing on deriving an optimal combination of hyperparameters with fewer iterations.

The proposed method was tested with CLD value of 3 to maintain the same environment as the existing method. Experiments were conducted using the same dataset used in a previous study using the optimal hyperparameter combination derived by the proposed method. As the result, the recognition rate of the 1D CNN’s respiration pattern was approximately 96.7%, which is an improvement of approximately 2.8% over the previous study.

In addition, to derive hyperparameters, the number of experimental repetitions was 2,000,000 with the existing method. In contrast, the proposed method could derive an optimal combination of hyperparameters in only 3652 iterations.

This demonstrates the excellence of the proposed method and suggests that the time required to derive an optimal combination of hyperparameters can be reduced by up to approximately 548 times compared to the existing method.

## Figures and Tables

**Table 1:** Specifications of UWB sensor.

Item	Specification
Detecting Range	10~22 (m)
Frequency Range	3.0~4.0 (GHz)
Bandwidth	0.45~1.0 (GHz)
Distance Resolution	1.5~3.3 (cm)
Antenna Angle	50° (X-Z plane)~77.5° (Y-Z plane)

**Table 2:** Respiration type according to number of respirations per minute.

Type of Respiration	Definition and Characteristics of Respiration
Eupnea	Respiration when average number of respirations per minute is 15–20 for an adult.
Bradypnea	Respiration when the number of respirations per minute is 12 or less. Compared to general respiration, the depth of inspiration and expiration is reduced, and the respiration cycle is increased. Often observed when sleeping and may be caused by diseases.
Tachypnea	Shallow respiration with 20 or more respirations per minute and may occur in the presence of diseases, e.g., fever and weakness or mental instability. This can be appeared during normal light exercise.
Apnea	When there is a decrease of more than 90% of typical respiratory airflow for more than 10 seconds during sleep. This results in very small amplitudes in the respiratory signal.

**Table 3:** Hyperparameters of general 1D CNN.

Hyperparameter	Description
Kernel Size	Kernel size of convolutional layer
Kernel Count	Kernel count of convolutional layer
Stride	Number of moving pixels of kernel when performing convolution (base: 1)
Zero-padding	Hyperparameters used to acquire characteristics of the border area of the training data
Epoch	Number of learning iterations
Learning Rate	Amount of change in weight that is updated during learning
Layer Depth	Number of layers constituting entire network
Neuron Count	Neuron count in fully-connected layer
Batch Size	Group size to divide training data into several groups
Loss Function	Function to calculate error (SGD)
Activation Function	Neuron’s activation function (ReLU, sigmoid, etc.)

**Table 4:** Hyperparameters to be optimized for 1D CNN.

Hyperparameter	Description	Expression of Hyperparameter in Layer
*KS*	Kernel size of each convolution layer	KS = {*ks^1^*, …, *ks^cld^*}
*KC*	Kernel count of each convolution layer	KC = {*kc^1^*, …, *kc^cld^*}
*DLNC*	Neuron count of each dense layer	DLNC = {*dlnc^1^*, *dlnc^2^*}

**Table 5:** Number of iterations required to reach 95% recognition rate for each hyperparameter combination to find appropriate hyperparameter value for HS algorithm.

Methods	Hyperparameters for HS Algorithm			Iteration	Set Recognition Rate (%)
	HMCR	PAR	MPAP		
Existing method 1 [38]	0.95	0.8	0.2	5912	95%
Existing method 2 [39]	0.70	0.50	0.1	4357	
Proposed method	0.5–0.7	0.6–0.8	10–18	2011	

**Table 6:** Samples of five respiration patterns collected from participants.

Pattern	Samples of Signal
Eupnea	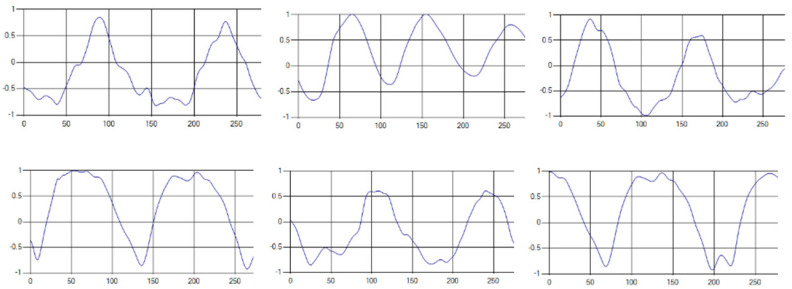
Bradypnea	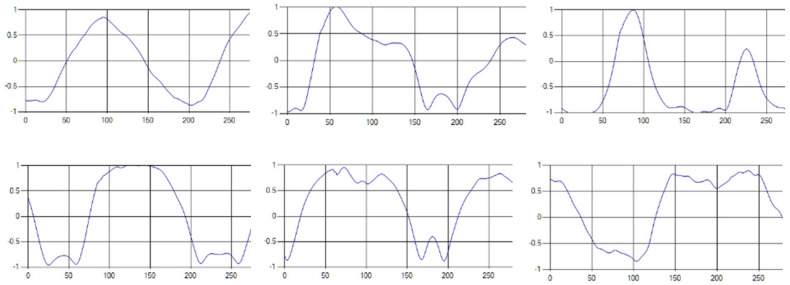
Tachypnea	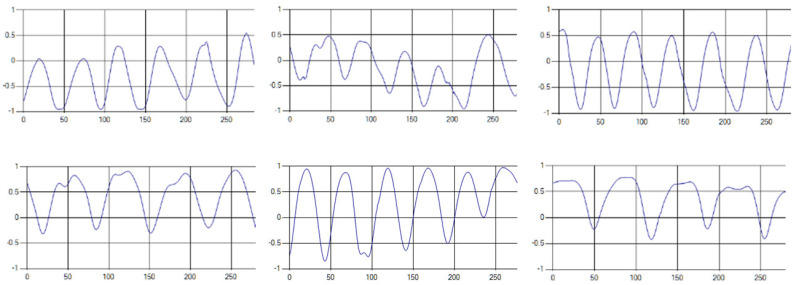
Apnea	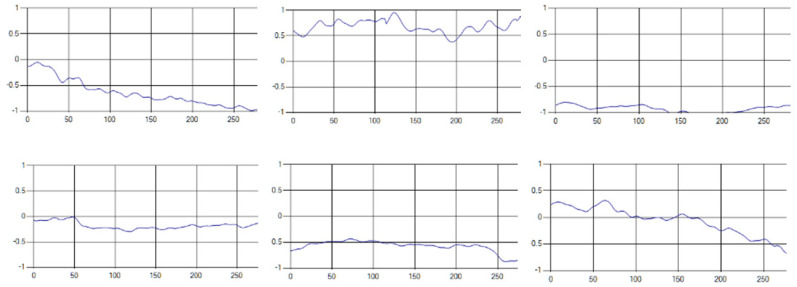
Moving	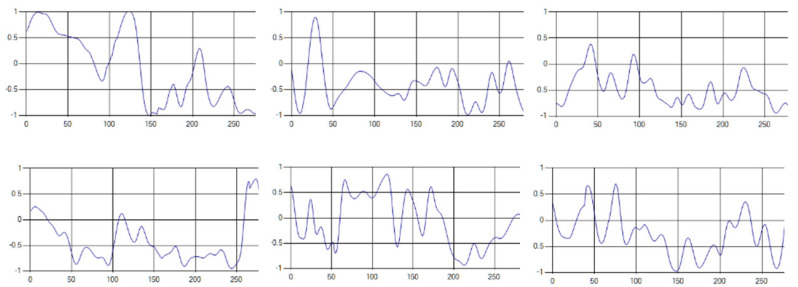

**Table 7:** Hyperparameters used in proposed method and range of each parameter value.

Parameter	Description	Value or Range
*HMS*	Harmony Memory Size	1000
*HMCR*	Harmony Memory Considering Ratio	0.5~0.7
*PAR*	Pitch Adjusting Ratio	0.6~0.8
*MPAP*	Maximum Pitch Adjustment Proportion	10~18
*Iteration*	Number of repetitions for HM update	10,000
*k*	Threshold for comparing the number ofnon-updates of $H_{max}$	200
*CLD*	Convolution Layer Depth	3
*KS^i^_i=1,2,3_*	Kernel Size for Convolution	3~81
*KC^i^_i=1,2,3_*	Kernel Count for Convolution	16~1024
*DLNC^1^*	First Dense Layer (FC Layer) Neuron Count	256~4096
*DLNC^2^*	Second Dense Layer (FC Layer) Neuron Count	256~4096

**Table 8:** Hyperparameter combination and recognition rate when $H_{max}$ was updated.

Iteration	Hyperparameters in HM								$\mathrm{Recognition}\;\mathrm{Rate}\;\mathrm{of}\;H_{max}\;$ (%)
	$ks^{1}$	$kc^{1}$	$ks^{2}$	$kc^{2}$	$ks^{3}$	$kc^{3}$	$dlnc^{1}$	$dlnc^{2}$	
1	5	171	63	314	27	573	3426	1796	83.4
5	19	481	5	56	21	214	862	2182	83.9
21	47	98	27	228	55	638	1544	3,205	84.5
55	37	116	73	187	9	203	929	648	84.6
⋮									
3652	23	56	17	48	11	102	1762	984	96.7

**Table 9:** Number of iterations required for hyperparameter optimization and recognition rate using optimized hyperparameters.

Method	Number ofIteration	Hyperparameters for HS Algorithm								Recognition Rate (%)
		$ks^{1}$	$ks^{2}$	$ks^{3}$	$kc^{1}$	$kc^{2}$	$kc^{3}$	$dlnc^{1}$	$dlnc^{2}$	
Previous Method [25]	2,000,000	$ks^{i}\in KS,\;when$ $KS=\left\{21,25,29\right\}$			$kc^{i}\in KC,\;when$ $KC=\left\{64,128\right\}$			$dlnc^{i}\in DLNC,when$ $DLNC=\left\{1024,\;2048\right\}$		93.9
Proposed Method	3652	23	17	11	56	48	102	1762	984	96.7
